# Defining the role of antioxidants in the prevention of prostate cancer

**Published:** 2009

**Authors:** Hemant R. Pathak, Bhupendra P. Singh

**Affiliations:** Department of Urology, BYL Nair Hospital and TN Medical College, Mumbai, India E-mail: hemantrpathak@gmail.com

## SUMMARY

This is the largest individually randomized, multicenter cancer prevention trial conducted that has maintained high rates of adherence and retention for 4 to 7 years. This trial recruited 35,533 men who were randomly assigned to four groups (selenium, vitamin E, selenium + vitamin E, and placebo) in a double-blind fashion between 2001 and 2004. Eligibility included age 50 years or older (African American men) or 55 years or older (all other men), a serum prostate-specific antigen level of ≤4 ng/ml, and a digital rectal examination not suspicious for prostate cancer. Antioxidant formulations used were oral selenium (200 μg/d from L-selenomethionine) and matched vitamin E placebo, vitamin E (400 IU/d of all rac-α-tocopheryl acetate), and matched selenium placebo, selenium + vitamin E, or placebo + placebo for a follow-up of minimum of 7 years and a maximum of 12 years. The primary outcome was prostate cancer and prespecified secondary outcomes were lung, colorectal, and overall primary cancer. At median overall follow-up of 5.46 years (range: 4.17–7.33 years), hazard ratios for prostate cancer were 1.13 (99% CI, 0.95–1.35; *n* = 473) for vitamin E, 1.04 (99% CI, 0.87–1.24; *n* = 432) for selenium, and 1.05 (99% CI, 0.88–1.25; *n* = 437) for selenium + vitamin E *vs.* 1.00 (*n* = 416) for placebo. No significant differences (all were *p* > 0.15) were seen in any other prespecified cancer endpoints. There were statistically non significant increased risks of prostate cancer in the vitamin E group (p = 0.06) and Type 2 diabetes mellitus in the selenium group (relative risk, 1.07; 99% CI, 0.94–1.22; p = 0.16), but not in the selenium + vitamin E group. This study concluded that selenium or vitamin E alone or in combination at the doses and formulations used did not prevent prostate cancer in this population of relatively healthy men.

## COMMENTS

There has been some data supporting the role of antioxidants and vitamins in preventing prostate cancer. Of these, the secondary outcomes of two randomized controlled trials were important: the Nutritional Prevention of Cancer (NPC) study[1,2] and the Alpha-Tocopherol, Beta-Carotene Cancer Prevention (ATBC) study,[3] which showed prostate cancer risk reductions of 63% for selenized yeast and 32% for α-tocopherol (vitamin E). In addition, a large-scale randomized controlled trial[4] involving several different regimens found that a combination of selenium, vitamin E, and beta carotene reduced overall cancer mortality. These clinical data were also supported by some epidemiologic and preclinical data. This led to the initiation of two major randomized trials: Physicians health survey[5] (PHS)-II in 1997 and SELECT in 2001. The results of these trials are disappointing with regard to prevention of prostate cancer by vitamin E, C, and selenium.

Further, one more cancer prevention trial[6] failed to show any benefit of supplementation with vitamin C, E, or beta carotene in the primary prevention of total cancer incidence or cancer mortality (not for the site-specific cancer). In a nutshell, the role of vitamin E was suggested by the ATBC study and the NPC study and was disproved by HOPE-TOO,[7] PHS-II, and SELECT studies. The role of vitamin C was disproved by the PHS-II study (for the first time in men, vitamin C is used for cancer prevention in a large trial) and the RCT study by Lin, *et al.* The role of selenium, which was suggested by ATBC study and the Chinese cancer prevention trial was disproved by the SELECT study. However, certain facts are to be kept in mind while drawing conclusions from these recent large randomized trials. First, these were conducted on a relatively healthy general population. Second, the dose of vitamin E used was much higher (400 IU/d) than that used in the ATBC study (50 IU/d). Third, the population of smokers was much less as compared with the ATBC study. Fourth, the SELECT study cannot comment on the effect on development of advanced cancer (Cancer Progression). Fifth, the duration of follow-up was limited to 4–8 years. Furthermore, the PHS-II and SELECT studies found vitamin E and C to be safer in used doses in contrast with earlier reports. Selenium alone was found to cause a statistically insignificant increase in DM-2 in the SELECT study similar to a recent analysis of the NPC study population.[8] Whether previous secondary outcomes on preventing cancer were by chance (which most likely is the possibility) or those results were specific to drug dose, specific group of population/patients/disease status is a debatable issue. However, a definite lesson learned from these sequential trials is that physicians should not recommend these antioxidants/vitamins to their patients for preventing prostate cancer until their definite causal role is proved by further generation trials.
